# Supplementary material for: Long-Term Consequences of COVID-19 at 6 Months and Above: A Systematic Review and Meta-Analysis
Source: Int J Environ Res Public Health. 2022 Jun 3;19(11):6865. doi: 10.3390/ijerph19116865 (PMC9180091; doi:10.3390/ijerph19116865)
Supplement: Supplementary file 1 [file ijerph-19-06865-s001.zip › ijerph-1728015-supplementary.pdf]

Supplementary Table S1. Template of primary data extraction

Content of data extraction	Notes
First author	-
Publication time	-
Study design	-
County where the study was Conducted	-
follow-up period (months)	-
Population size	-
Sex ratio (Male/Female)	-
Age	-
Smoker (n/N)	-
Degree of severity of COVID-19 (n/N)	-
≥1 comorbidity (n/N) and types of underlying conditions (n)	-
Number of hospitalization (n/N) and length of stay (days)	-
Number of ICU (n/N) and length of stay (days)	-
Number of death (n/N)	-
Types of respiratory support (n)	-
General symptoms (n/N)	including ≥1 symptoms, fever, chill, fatigue, muscle weakness, fatigue or muscle weakness, limited mobility, myalgia, joint pain, myalgia or joint pain, headache, dizzness, taste loss, olfactory loss, olfactory or taste loss, hair loss, cutaneous
Respiratory symptoms	including cough, expectoration, shortness of breath, rhinorrhea, sore throat or difficult to swallow, dyspnea, mMRC-0, mMRC ≥1
Cardiovascular symptoms	including chest tightness, chest pain, back pain, palpitations
Gastrointestinal symptoms	including GI symptoms, loss of appetite, nausea or vomiting or diarrhoe, vomiting, nause, diarrhoea, stomachache, constipation
Neurological symptoms	including neurological symptoms, polyneuropathy, paresthesias, disorientation or confusion, forgetfulness, memory loss, visual impairment, hearing impairment
Psychiatric symptoms	including sleep difficulty, GAD-7, depression, anxiety, PTSD, difficulty concentrating
serology	including IgM positive, IgG positive
Pulmonary functional test	including FVC＜80%, FEV1＜80%, FEV1/FEV＜70%, VC＜80%, TLC＜80%, DLCO＜80%
CT results	including CT abnormity, bilateral ditribution, GGO, consolidation, reticular pattern, fibrosis, crazy paving pattern, air bronchogram bronchiectasis, traction bronchiectasis, nodules, irregular interface, parenchymal band, pleural effusion, pericardial effusion, lymphadenopathy, Interlobular spetal thickening, lines and bands
Quality of life evaluation (EQ-5D-5L)	including mobility, personal care, usual activity, pain or discomfort, anxiety and depression, 6MWT (distance lower than expected %）

Supplementary Table S2. Characteristic of the included studies.

Record number	The first author	Publication time	Country	Follow-up time	Total number (N)	Gender male/female	Age	Smoker (n/N)	Degree of severity (n）	At least one comorbidity (n/N)	Types of comorbidities (n)	Number of hospitalizations (n/N)	Length of stay (days)	Number of ICU admission (n/N)	Length of ICU stay (days)	death
1	Liqun Huang	2022.1	China	6 months	574	226/348	57.7	-	113 severe cases	245/574	156 hypertension, 84 diabetes, 37 coronary heart disease, 22 chronic lung disease, 7 chronic kidney disease, 12 chronic liver disease, 13 cerebrovascular disease, 16 cancer	574/574	18.49	-	-	0
2	Lindahl, A.	2021.8	Finland	6 months	101	54/47	60.0	46/101	-	70/101	31 hypertension, 19 asthma, 19 hyperlipidaemia, 13 diabetes, 9 coronary artery disease, 7 hypothyroidism, 6 cancer, 6 heart arrhythmia, 5 neurological disorders	101/101	15.00	34/101	15	0
3	Mazza, M. G.	2022.01	Italy	6.5 months 12 months	216	150/66	60.1	-	-	-	-	216/216	-	23/216	-	0
4	Romero-Duarte, Á	2021.05	Spain	6 months	797	428/369	-	-	-	538/797	409 hypertension, 166 diabetes, 164 cardiovascular disease, 107 pneumopathy, 40 COPD, 59 asthma, 69 chronic kidney disease, 61 autoimmune disease, 36 immunosuppression, 32 active neoplasms	85/797	15.00	81/797	-	0
5	Xiong, L.	2021.07	China	8 months	333	76/257	36.0	7/333	0 mild or moderate cases, 333 severe cases	103/333	-	333/333	-	17/328	-	0
6	Faverio, P.	2021.11	Italy	6 months	312	229/83	61.1	80/312	-	181/312	34% obesity, 29% hypertension, 22% cardiovascular diseases, 14% diabetes	312/312	-	-	-	0
7	Eloy, P.	2021.11	France	6 months	324	205/119	64.0	94/265	-	312/311	48 diabetes, 110 hypertension, 53 obesity, 57 chronic cardiac disease, 56 chronic pulmonary disease, 16 chronic kidney disease, 3 moderate or severe chronic liver disease, 4 mild chronic liver disease, 20 chronic neurological disorder, 20 malignant neoplasm, 16 chronic haematologic disease, 1 AIDS/HIV, 1 dementia,16 rheumatologic disorder	324/324	-	54/286	-	0

8	Caruso, D.	2021.07	Italy	6 months	118	56/62	65.0	51/118	118 moderate or severe cases	82/118	40 hypertension, 18 cardiovascular disease, 11 diabetes, 13 others	118/118	-	-	-	0
9	Peghin, M.	2021.10	Italy	6 months	599	279/320	53.0	-	-	314/599	135 hypertension, 98 obesity, 33 diabetes, 21 chronic respiratory disease, 7 cardiovascular disease, 10 liver disease, 6 psychiatric disorders	157/599	7.00	23/599	-	0
10	Liu, M.	2021.09	China	6 months	52	26/26	50.5	-	32 moderate cases, 20 severe cases	-	6 diabetes, 7 hypertension, 2 COPD, 2 cardiovascular disease,1 liver disease	52/52	17.00	-	-	0
11	Nehme, M.	2021.09	Geneva	7-9 months	410	135/275	42.7	-	-	113/410	11 cardiovascular disease, 30 hypertension, 23 chronic respiratory disease, 12 diabetes, 7 cancer, 4 immunosuppression, 10 other chronic disease	0/410	0.00	-	-	0
12	Huang, L.	2021.08	China	6 months, 12 months	1276	681/595	59.0	225/1276	-	-	-	1276/1276	14.00	54/1276	18.00	0
13	Darcis, G.	2021.08	Belgium	6 months	199	126/73	60.5	53/196	-	-	-	199/199	9	52/199	-	0
14	Xiao, K.	2021.07	China	6 months, 12 months	35	-	-	-	24 mild or moderate cases, 11 severe cases	-	-	35/35	-	-	-	0
15	Wu, X.	2021.07	China	6 months, 12 months	83	47/36	60.0	0/83	83 severe cases	0/83	none	83/83	29.00	-	-	0
16	Menges, D.	2021.07	Switzerland	6-8 months	431	217/214	47.0	183/428	46 asymptomatic cases, 221 mild or moderate cases, 164 severe cases	147/430	-	71/431	-	10/431	-	0
17	Fayol, A.	2021.07	France	6 months	48	33/15	58.0	-	-	32/48	17 hypertension, 10 diabetes, 14 hypercholesterolemia, 3 prior myocardial infarction, 1 prior valvular disease	48/48	-	13/48	-	0
18	Han, X.	2021.01	China	6 months	114	80/34	54.0	16/114	-	59/114	13 diabetes, 32 hypertension, 16 chronic pulmonary disease	114/114	17.00	-	-	0
19	Liu, M.	2021.03	China	7 months	41	22/19	50.0	-	26 moderate cases, 15 severe cases	7/41	1 diabetes, 3 hypertension, 2 COPD, 1 cardiovascular disease, 1 hepatic disease	41/41	18.00	-	-	0
20	Huang, C.	2021.01	China	6 months	1733	897/836	57.0	146/1731	-	-	-	1733/1733	14.00	76/1733	14.00	0
21	Bai, Tao	2021.12	China	6 months	40	17/23	55.1	7/40	14 mild cases, 26 severe cases	-	5 cardiopulmonary diseases, 14 heart disease, 12 hyperlipidaemia, 6 diabetes	40/40	-	-	-	0

22	Augustin, Max	2021.07	Germany	6.8 months	353	151/202	-	-	-	-	-	0/353	-	-	-	0
23	van Veenendaal, Nadine	2021.07	Netherlands	6 months	60	41/19	62.5	-	60 severe cases	-	-	60/60	30.60	60/60	19.40	0
24	Peluso, Michael J.	2021.12	America	8 months	179	98/78	48.0	-	-	-	-	44/179	-	-	-	0
25	Erber, Johanna	2021.11	Germany	7 months	18	14/4	54.0	7/18	-	13/18	8 hypertension, 4 diabetes, 4 adipositas, 1 coronary heart disease, 2 COPD, 1 asthma bronchiale, 4 chronic kidney disease, 2 chronic liver disease, 3 cancer, 1 HIV, 3 immunosuppression	18/18	21.50	18/18	10.00	0
26	Kim, Y.	2022.01	Korea	6 months, 12 months	241	77/164	37.0	-	11 asymptomatic cases, 194 mild cases, 30 moderate cases, 6 severe cases	-	-	132/241	-	-	-	0
27	Yan-Yao Du	2021.12	China	12 months	19	8/11	54.2	2/19	10 mild cases, 9 severe cases	-	-	19/19	19.2	-	-	0
28	Tessitore, E.	2021.12	Geneva	12 months	184	114/74	58.0	-	-	-	65 hypertension, 45 dyslipidemia, 29 diabetes, 2 congestive heart failure	184/184	11.1	29/184	-	0
29	Zhan, Y.	2021.10	China	12 months	121	50/71	49.0	-	102 mild or moderate cases, 19 severe cases	37/121	31 hypertension, 8 diabetes, 2 autoimmune diseases, 3 cardiovascular diseases, 1 cancer	121/121	-	-	-	0
30	Mainous, A. G.	2021.12	America	12 months	424	162/262	70.5	-	246 mild or moderate cases, 178 severe cases	76/162	coronary artery disease, heart failure, valvulopathy (grade ≥ moderate), cancer, COPD. asthma	-	-	-	-	0
31	Zhou, F.	2021.11	China	12 months	120	49/71	51.6	16/120	104 mild or moderate cases, 16 severe cases	-	6 diabetes, 20 hypertension, 4 hyperlipemia, 3 coronary heart disease	120/120	25.5	-	-	0
32	Bellan, M.	2021.11		12 months	200	122/78	62.0	99/200	-		82 arterial hypertension, 31 diabetes, 18 dyslipidemia, 12 COPD, 22 obesity, 4 IBD, 7 chronic liver disease, 3 autoimmune disease, 13 hematological disease, 18 coronary artery disease, 13 atrial fibrillation,3 other structural heart disease, 6 other arrhythmogenic heart disease, 22 endocrinological disease, 12 CKD, 5 stroke/TIA, 4	200/200	9.0	23/200	10.0	0

											VTE, 8 anxiety and depression, 18 active malignancy					
33	Zhao, Y.	2021.11	China	12 months	94	54/40	48.1	7/94	-	-	16 hypertension, 9 diabetes, 4 chronic heart disease, 2 asthma	94/94	15.1	11/94	-	0
34	Maestrini, V.	2021.09		12 months	424	-	-	-	246 mild or moderate cases, 178 severe cases	227/424	-	-	-	-	-	0
35	Lombardo, M. D. M.	2021.08		12 months	303	138/165	53.0	111/303	-	-	89 hypertension, 32 cardiovascular disease, 28 diabetes, 18 cancer, 17 COPD, 11 cerebrovascular disease, 4 chronic kidney disease	189/303	-	8/303	-	0
36	Xianyu, Yunyan	2022.01	China	12 months	18	0/18	31.5	0/18	3 mild cases, 15 moderate cases	-	3 gestational hypertension, 2 diabetes, 1 thyroid disease, 1 chronic kidney disease	18/18	10.0	0/18	-	0
37	Gamberini, Lorenzo	2021.11	Italy	12 months	178	129/49	64.0	-	93 mild or moderate cases, 78 severe cases	-	88 hypertension, 13 chronic ischemic heart disease, 6 chronic kidney disease, 13 COPD	178/178	23.0	178/178	23.0	0
38	Becker, Christoph	2021.10	Switzerland	12 months	90	57/34	60.1	-	-	-	-	90/90	9.4	15/90	-	0
39	Molhave, Martin	2021.11	Denmark	12 months	45	21/24	57.0	18/45	-	28/45	13 hypertension, 13 asthma, 4 coronary heart disease, 4 malignancy, 3 COPD, 1 transplanted, 6 inflammatory diseases, 3 hypercholesterolemia, 1 arthritis urica, 1 psoriasis, 1 hypothyroidism	45/45	7.0	8/45	-	0
40	Fortini, A.	2022.01		12 months	17	8/9	71.0	-	-	-	-	17/17	-	0/17	-	0

Supplementary Table S3. Risk of bias and quality of included studies assessed by Newcastle-Ottawa quality assessment Scale (NOS).

		Selection				Comparabilty	Outcome				
Record number	The first author	Representativeness of the exposed cohort	Selection of the non-exposed cohort	Ascertainment of exposure	Demonstration that outcome of interest was not present at start of study	Comparability of cohorts on basis of the design or analysis	Assessment of outcome	Was follow-up long enough for outcome to occur	Adequeacy of follow up of cohorts	Quality score	The risk of bias
1	Liqun Huang	1	1	1	1	1	1	1	1	8	Low
2	Lindahl, A.	1	1	1	1	1	1	1	1	8	Low
3	Mazza, M. G.	1	1	1	1	1	1	1	1	8	Low
4	Romero-Duarte, Á	1	1	1	1	1	1	1	1	8	Low
5	Xiong, L.	0	1	1	1	0	1	1	1	6	moderate
6	Faverio, P.	1	1	1	1	2	1	1	1	9	Low
7	Eloy, P.	1	1	1	1	0	1	1	1	7	Low
8	Caruso, D.	1	1	1	1	2	1	1	1	9	Low
9	Peghin, M.	1	1	1	1	2	1	1	1	9	Low
10	Liu, M.	1	1	1	1	0	1	1	1	7	Low
11	Nehme, M.	1	1	1	1	0	0	1	1	6	moderate
12	Huang, L.	1	1	1	1	2	1	1	1	9	Low
13	Darcis, G.	1	1	1	1	2	1	1	1	9	Low
14	Xiao, K.	1	1	1	1	0	1	1	1	7	Low
15	Wu, X.	1	1	1	1	2	1	1	1	9	Low
16	Menges, D.	1	1	1	1	2	1	1	1	9	Low
17	Fayol, A.	1	1	1	1	0	1	1	1	7	Low
18	Han, X.	1	1	1	1	2	1	1	1	9	Low
19	Liu, M.	1	1	1	1	2	1	1	1	9	Low
20	Huang, C.	1	1	1	1	2	1	1	1	9	Low
21	Bai, Tao	1	1	1	1	0	1	1	1	7	Low
22	Augustin, Max	1	1	1	1	2	1	1	1	9	Low
23	van Veenendaal,	1	1	1	1	0	1	1	1	7	Low

	Nadine										
24	Peluso, Michael J.	1	1	1	1	0	1	1	1	7	Low
25	Erber, Johanna	1	1	1	1	0	1	1	1	7	Low
26	Kim, Y.	1	1	1	1	2	1	1	1	9	Low
27	Yan-Yao Du	1	1	1	1	0	1	1	1	7	Low
28	Tessitore, E.	1	1	1	1	0	1	1	1	7	Low
29	Zhan, Y.	1	1	1	1	2	1	1	1	9	Low
30	Mainous, A. G.	1	1	1	1	2	1	1	1	9	Low
31	Zhou, F.	1	1	1	1	0	1	1	1	7	Low
32	Bellan, M.	1	1	1	1	1	1	1	1	8	Low
33	Zhao, Y.	1	1	1	1	2	1	1	1	9	Low
34	Maestrini, V.	1	1	1	1	2	1	1	1	9	Low
35	Lombardo, M. D. M.	1	1	1	1	2	1	1	1	9	Low
36	Xianyu, Yunyan	1	1	1	1	0	1	1	1	7	Low
37	Gamberini, Lorenzo	1	1	1	1	2	1	1	1	9	Low
38	Becker, Christoph	1	1	1	1	2	1	1	1	9	Low
39	Molhave, Martin	1	1	1	1	2	1	1	1	9	Low
40	Fortini, A.	1	1	1	1	0	1	1	1	7	Low

Supplementary Table S4. GRADE evidence evaluation results of COVID-19 consequences in 6-12 months follow-up.

Consequence	No. of studies	Design	Quality assessment					Upgrade factors	Quality	Importance
			Risk of bias	Inconsistency	Indirectness	Imprecision	Publication bias			
**General symptoms**										
≥ 1 symptom	13	observational cohort study	no serious risk of bias	serious	no serious indirectness	no serious imprecision	undetected	Large magnitude of an effect, confounders likely minimize the effect	ÅÅÅÅ HIGH	CRITICAL
Fever	7	observational cohort study	no serious risk of bias	serious	no serious indirectness	no serious imprecision	undetected	none	ÅÅ LOW	IMPORTANT
Chill	2	observational cohort study	no serious risk of bias	serious	no serious indirectness	no serious imprecision	undetected	none	ÅÅ LOW	IMPORTANT
Fatigue	10	observational cohort study	no serious risk of bias	serious	no serious indirectness	no serious imprecision	undetected	Large magnitude of an effect, confounders likely minimize the effect	ÅÅÅÅ HIGH	CRITICAL
Muscle weakness	2	observational cohort study	no serious risk of bias	no serious inconsistency	no serious indirectness	no serious imprecision	undetected	none	ÅÅ LOW	IMPORTANT
Fatigue or muscle weakness	3	observational cohort study	no serious risk of bias	serious	no serious indirectness	no serious imprecision	undetected	Large magnitude of an effect	ÅÅÅ MODERATE	IMPORTANT
Limited mobility	3	observational cohort study	no serious risk of bias	serious	no serious indirectness	no serious imprecision	undetected	none	ÅÅ LOW	IMPORTANT

Myalgia	9	observational cohort study	no serious risk of bias	serious	no serious indirectness	no serious imprecision	undetected	confounders likely minimize the effect	ÅÅÅ MODERATE	IMPORTANT
Joint pain	6	observational cohort study	no serious risk of bias	serious	no serious indirectness	no serious imprecision	undetected	none	ÅÅ LOW	IMPORTANT
Headache	8	observational cohort study	no serious risk of bias	serious	no serious indirectness	no serious imprecision	undetected	confounders likely minimize the effect	ÅÅÅ MODERATE	IMPORTANT
Dizziness	5	observational cohort study	no serious risk of bias	serious	no serious indirectness	no serious imprecision	undetected	none	ÅÅ LOW	IMPORTANT
Olfactory or taste loss	4	observational cohort study	no serious risk of bias	serious	no serious indirectness	no serious imprecision	undetected	none	ÅÅ LOW	IMPORTANT
Olfactory loss	8	observational cohort study	no serious risk of bias	serious	no serious indirectness	no serious imprecision	undetected	confounders likely minimize the effect	ÅÅÅ MODERATE	IMPORTANT
Taste loss	8	observational cohort study	no serious risk of bias	serious	no serious indirectness	no serious imprecision	undetected	confounders likely minimize the effect	ÅÅÅ MODERATE	IMPORTANT
Hair loss	7	observational cohort study	no serious risk of bias	serious	no serious indirectness	no serious imprecision	undetected	confounders likely minimize the effect	ÅÅÅ MODERATE	IMPORTANT
Cutaneous	7	observational cohort study	no serious risk of bias	serious	no serious indirectness	no serious imprecision	undetected	none	ÅÅ LOW	IMPORTANT
**Respiratory symptoms**										

Cough	12	observational cohort study	no serious risk of bias	serious	no serious indirectness	no serious imprecision	undetected	confounders likely minimize the effect	ÅÅÅ MODERATE	IMPORTANT
Expectoration	3	observational cohort study	no serious risk of bias	serious	no serious indirectness	no serious imprecision	undetected	none	ÅÅ LOW	IMPORTANT
Rhinorrhea	2	observational cohort study	no serious risk of bias	serious	no serious indirectness	no serious imprecision	undetected	none	ÅÅ LOW	IMPORTANT
Sore throat or difficult to swallow	7	observational cohort study	no serious risk of bias	serious	no serious indirectness	no serious imprecision	undetected	none	ÅÅ LOW	IMPORTANT
Dyspnea	12	observational cohort study	no serious risk of bias	serious	no serious indirectness	no serious imprecision	undetected	confounders likely minimize the effect	ÅÅÅ MODERATE	IMPORTANT
mMRC = 0	5	observational cohort study	no serious risk of bias	serious	no serious indirectness	no serious imprecision	undetected	Very large magnitude of an effect; confounders likely minimize the effect	ÅÅÅÅ HIGH	CRITICAL
mMRC ≥ 1	5	observational cohort study	no serious risk of bias	serious	no serious indirectness	no serious imprecision	undetected	none	ÅÅ LOW	IMPORTANT
**Cardiovascular symptoms**										
Chest tightness	2	observational cohort study	no serious risk of bias	serious	no serious indirectness	no serious imprecision	undetected	none	ÅÅ LOW	IMPORTANT
Chest pain	9	observational cohort study	no serious risk of bias	serious	no serious indirectness	no serious imprecision	undetected	confounders likely minimize the	ÅÅÅ MODERATE	IMPORTANT

								effect		
Back pain	2	observational cohort study	no serious risk of bias	serious	no serious indirectness	no serious imprecision	undetected	none	ÅÅ LOW	IMPORTANT
Palpitations	5	observational cohort study	no serious risk of bias	serious	no serious indirectness	no serious imprecision	undetected	none	ÅÅ LOW	IMPORTANT
**Gastrointestinal symptoms**										
GI symptoms	4	observational cohort study	no serious risk of bias	serious	no serious indirectness	no serious imprecision	undetected	none	ÅÅ LOW	IMPORTANT
Loss of appetite	7	observational cohort study	no serious risk of bias	serious	no serious indirectness	no serious imprecision	undetected	confounders likely minimize the effect	ÅÅÅ MODERATE	IMPORTANT
Nausea, vomiting or diarrhoea	4	observational cohort study	no serious risk of bias	serious	no serious indirectness	no serious imprecision	undetected	none	ÅÅ LOW	IMPORTANT
Vomiting	2	observational cohort study	no serious risk of bias	no serious inconsistency	no serious indirectness	no serious imprecision	undetected	none	ÅÅ LOW	IMPORTANT
Diarrhoea	8	observational cohort study	no serious risk of bias	serious	no serious indirectness	no serious imprecision	undetected	confounders likely minimize the effect	ÅÅÅ MODERATE	IMPORTANT
Stomachache	2	observational cohort study	no serious risk of bias	serious	no serious indirectness	no serious imprecision	undetected	none	ÅÅ LOW	IMPORTANT
Constipation	2	observational cohort study	no serious risk of bias	serious	no serious indirectness	no serious imprecision	undetected	none	ÅÅ LOW	IMPORTANT
**Neurological symptoms**										
Neurological symptoms	3	observational cohort study	no serious risk of bias	serious	no serious indirectness	no serious imprecision	undetected	none	ÅÅ LOW	IMPORTANT
Polyneuropathy	2	observational cohort study	no serious risk of bias	serious	no serious indirectness	no serious imprecision	undetected	none	ÅÅ LOW	IMPORTANT

Paresthesias	4	observational cohort study	no serious risk of bias	serious	no serious indirectness	no serious imprecision	undetected	none	ÅÅ LOW	IMPORTANT
Disorientation or confusion	3	observational cohort study	no serious risk of bias	serious	no serious indirectness	no serious imprecision	undetected	none	ÅÅ LOW	IMPORTANT
Forgetfulness	2	observational cohort study	no serious risk of bias	serious	no serious indirectness	no serious imprecision	undetected	none	ÅÅ LOW	IMPORTANT
Memory loss	3	observational cohort study	no serious risk of bias	serious	no serious indirectness	no serious imprecision	undetected	none	ÅÅ LOW	IMPORTANT
Visual impairment	3	observational cohort study	no serious risk of bias	serious	no serious indirectness	no serious imprecision	undetected	none	ÅÅ LOW	IMPORTANT
Hearing impairment	2	observational cohort study	no serious risk of bias	no serious inconsistency	no serious indirectness	no serious imprecision	undetected	none	ÅÅ LOW	IMPORTANT
**Psychiatric symptoms**										
Sleep difficulty	9	observational cohort study	no serious risk of bias	serious	no serious indirectness	no serious imprecision	undetected	confounders likely minimize the effect	ÅÅÅ MODERATE	IMPORTANT
GAD-7 score ≥ 10	2	observational cohort study	no serious risk of bias	serious	no serious indirectness	no serious imprecision	undetected	none	ÅÅ LOW	IMPORTANT
Depression	6	observational cohort study	no serious risk of bias	serious	no serious indirectness	no serious imprecision	undetected	confounders likely minimize the effect	ÅÅÅ MODERATE	CRITICAL
Anxiety	6	observational cohort study	no serious risk of bias	serious	no serious indirectness	no serious imprecision	undetected	confounders likely minimize the effect	ÅÅÅ MODERATE	CRITICAL
PTSD	3	observational	no serious	serious	no serious	no serious	undetected	none	ÅÅ	IMPORTANT

		cohort study	risk of bias		indirectness	imprecision			LOW	
Difficulty concentrating	3	observational cohort study	no serious risk of bias	serious	no serious indirectness	no serious imprecision	undetected	none	ÅÅ LOW	IMPORTANT
**PFT**										
FVC < 80%	4	observational cohort study	no serious risk of bias	serious	no serious indirectness	no serious imprecision	undetected	none	ÅÅ LOW	IMPORTANT
FEV1 < 80%	2	observational cohort study	no serious risk of bias	serious	no serious indirectness	no serious imprecision	undetected	none	ÅÅ LOW	IMPORTANT
FEV1/FEV < 70%	2	observational cohort study	no serious risk of bias	-	no serious indirectness	no serious imprecision	undetected	none	ÅÅ LOW	IMPORTANT
VC < 80%	2	observational cohort study	no serious risk of bias	no serious inconsistency	no serious indirectness	no serious imprecision	undetected	none	ÅÅ LOW	IMPORTANT
DLCO < 80%	4	observational cohort study	no serious risk of bias	serious	no serious indirectness	no serious imprecision	undetected	Large magnitude of an effect	ÅÅÅ MODERATE	IMPORTANT
**CT results**										
CT abnormality	4	observational cohort study	no serious risk of bias	serious	no serious indirectness	no serious imprecision	undetected	Large magnitude of an effect	ÅÅÅ MODERATE	IMPORTANT
GGO	5	observational cohort study	no serious risk of bias	serious	no serious indirectness	no serious imprecision	undetected	none	ÅÅ LOW	IMPORTANT
Consolidation	4	observational cohort study	no serious risk of bias	no serious inconsistency	no serious indirectness	no serious imprecision	undetected	none	ÅÅ LOW	IMPORTANT
Reticular pattern	4	observational cohort study	no serious risk of bias	serious	no serious indirectness	no serious imprecision	undetected	none	ÅÅ LOW	IMPORTANT
Fibrosis	3	observational cohort study	no serious risk of bias	serious	no serious indirectness	no serious imprecision	undetected	Large magnitude of an effect	ÅÅÅ MODERATE	IMPORTANT
Crazy paving	3	observational	no serious	-	no serious	no serious	undetected	none	ÅÅ	IMPORTANT

pattern		cohort study	risk of bias		indirectness	imprecision			LOW	
Air bronchogram	3	observational cohort study	no serious risk of bias	-	no serious indirectness	no serious imprecision	undetected	none	ÅÅ LOW	IMPORTANT
Bronchiectasis	3	observational cohort study	no serious risk of bias	serious	no serious indirectness	no serious imprecision	undetected	none	ÅÅ LOW	IMPORTANT
Traction bronchiectasis	2	observational cohort study	no serious risk of bias	serious	no serious indirectness	no serious imprecision	undetected	none	ÅÅ LOW	IMPORTANT
Nodules	2	observational cohort study	no serious risk of bias	serious	no serious indirectness	no serious imprecision	undetected	none	ÅÅ LOW	IMPORTANT
Irregular interface	2	observational cohort study	no serious risk of bias	no serious inconsistency	no serious indirectness	no serious imprecision	undetected	none	ÅÅ LOW	IMPORTANT
Parenchymal band	2	observational cohort study	no serious risk of bias	serious	no serious indirectness	no serious imprecision	undetected	none	ÅÅ LW	IMPORTANT
Pleural effusion	3	observational cohort study	no serious risk of bias	-	no serious indirectness	no serious imprecision	undetected	none	ÅÅ LOW	IMPORTANT
Pericardial effusion	2	observational cohort study	no serious risk of bias	-	no serious indirectness	no serious imprecision	undetected	none	ÅÅ LOW	IMPORTANT
Lymphadenopathy	2	observational cohort study	no serious risk of bias	-	no serious indirectness	no serious imprecision	undetected	none	ÅÅ LOW	IMPORTANT
Interlobular spetal thickening	4	observational cohort study	no serious risk of bias	serious	no serious indirectness	no serious imprecision	undetected	none	ÅÅ LOW	IMPORTANT
**Quality of life evaluation (EQ-5D-5L)**										
Mobility	2	observational cohort study	no serious risk of bias	serious	no serious indirectness	no serious imprecision	undetected	none	ÅÅ LOW	IMPORTANT
Personal care	2	observational cohort study	no serious risk of bias	serious	no serious indirectness	no serious imprecision	undetected	none	ÅÅ LOW	IMPORTANT
Usual activity	3	observational cohort study	no serious risk of bias	serious	no serious indirectness	no serious imprecision	undetected	none	ÅÅ LOW	IMPORTANT
Pain or discomfort	2	observational cohort study	no serious risk of bias	serious	no serious indirectness	no serious imprecision	undetected	Large magnitude of	ÅÅÅ MODERATE	CRITICAL

								an effect		
Anxiety and depression	2	observational cohort study	no serious risk of bias	serious	no serious indirectness	no serious imprecision	undetected	Large magnitude of an effect	ÅÅÅ MODERATE	CRITICAL
6MWT (distance lower than expected %）	4	observational cohort study	no serious risk of bias	serious	no serious indirectness	no serious imprecision	undetected	none	ÅÅ LOW	IMPORTANT

Supplementary Table S5. GRADE evidence evaluation results of COVID-19 consequences in 12 months and above follow-up.

Consequence	No. of studies	Design	Quality assessment					Upgrade factors	Quality	Importance
			Risk of bias	Inconsistency	Indirectness	Imprecision	Publication bias			
**General symptoms**										
≥ 1 symptom	8	observational cohort study	no serious risk of bias	serious	no serious indirectness	no serious imprecision	undetected	Large magnitude of an effect, confounders likely minimize the effect	ÅÅÅÅ HIGH	CRITICAL
Fever	7	observational cohort study	no serious risk of bias	serious	no serious indirectness	no serious imprecision	undetected	none	ÅÅ LOW	IMPORTANT
Fatigue	14	observational cohort study	no serious risk of bias	serious	no serious indirectness	no serious imprecision	undetected	Large magnitude of an effect, confounders likely minimize the effect	ÅÅÅÅ HIGH	CRITICAL
Myalgia or joint pain	2	observational cohort study	no serious risk of bias	serious	no serious indirectness	no serious imprecision	undetected	Large magnitude of an effect	ÅÅÅ MODERATE	IMPORTANT
Myalgia	9	observational cohort study	no serious risk of bias	serious	no serious indirectness	no serious imprecision	undetected	confounders likely minimize the effect	ÅÅÅ MODERATE	IMPORTANT
Joint pain	8	observational	no serious	serious	no serious	no serious	undetected	confounders	ÅÅÅ	IMPORTA

		cohort study	risk of bias		indirectness	imprecision		likely minimize the effect	MODERATE	NT
Headache	5	observational cohort study	no serious risk of bias	no serious inconsistency	no serious indirectness	no serious imprecision	undetected	none	ÅÅ LOW	IMPORTA NT
Dizziness	3	observational cohort study	no serious risk of bias	serious	no serious indirectness	no serious imprecision	undetected	none	ÅÅ LOW	IMPORTA NT
Olfactory or taste loss	2	observational cohort study	no serious risk of bias	serious	no serious indirectness	no serious imprecision	undetected	none	ÅÅ LOW	IMPORTA NT
Olfactory loss	8	observational cohort study	no serious risk of bias	serious	no serious indirectness	no serious imprecision	undetected	confounders likely minimize the effect	ÅÅÅ MODERATE	IMPORTA NT
Taste loss	7	observational cohort study	no serious risk of bias	serious	no serious indirectness	no serious imprecision	undetected	confounders likely minimize the effect	ÅÅÅ MODERATE	IMPORTA NT
Hair loss	4	observational cohort study	no serious risk of bias	serious	no serious indirectness	no serious imprecision	undetected	none	ÅÅ LOW	IMPORTA NT
Cutaneous	5	observational cohort study	no serious risk of bias	no serious inconsistency	no serious indirectness	no serious imprecision	undetected	none	ÅÅ LOW	IMPORTA NT
**Respiratory symptoms**										
Respiratory symptoms	2	observational cohort study	no serious risk of bias	serious	no serious indirectness	no serious imprecision	undetected	Large magnitude of an effect	ÅÅÅ MODERATE	IMPORTA NT
Cough	9	observational cohort study	no serious risk of bias	serious	no serious indirectness	no serious imprecision	undetected	confounders likely minimize the effect	ÅÅÅ MODERATE	IMPORTA NT
Expectoration	4	observational	no serious	serious	no serious	no serious	undetected	none	ÅÅ	IMPORTA

		cohort study	risk of bias		indirectness	imprecision			LOW	NT
Rhinorrhea	2	observational cohort study	no serious risk of bias	serious	no serious indirectness	no serious imprecision	undetected	none	ÅÅ LOW	IMPORTANT
Sore throat or difficult to swallow	6	observational cohort study	no serious risk of bias	serious	no serious indirectness	no serious imprecision	undetected	none	ÅÅ LOW	IMPORTANT
Dyspnea	8	observational cohort study	no serious risk of bias	serious	no serious indirectness	no serious imprecision	undetected	confounders likely minimize the effect	ÅÅÅ MODERATE	IMPORTANT
mMRC = 0	3	observational cohort study	no serious risk of bias	serious	no serious indirectness	no serious imprecision	undetected	Very large magnitude of an effect	ÅÅÅÅ HIGH	CRITICAL
mMRC ≥ 1	4	observational cohort study	no serious risk of bias	serious	no serious indirectness	no serious imprecision	undetected	none	ÅÅ LOW	IMPORTANT
**Cardiovascular symptoms**										
Chest tightness	3	observational cohort study	no serious risk of bias	serious	no serious indirectness	no serious imprecision	undetected	none	ÅÅ LOW	IMPORTANT
Chest pain	5	observational cohort study	no serious risk of bias	serious	no serious indirectness	no serious imprecision	undetected	none	ÅÅ LOW	IMPORTANT
Palpitations	7	observational cohort study	no serious risk of bias	serious	no serious indirectness	no serious imprecision	undetected	confounders likely minimize the effect	ÅÅÅ MODERATE	IMPORTANT
**Gastrointestinal symptoms**										
GI symptoms	5	observational cohort study	no serious risk of bias	serious	no serious indirectness	no serious imprecision	undetected	none	ÅÅ LOW	IMPORTANT
Loss of appetite	5	observational cohort study	no serious risk of bias	no serious inconsistency	no serious indirectness	no serious imprecision	undetected	none	ÅÅ LOW	IMPORTANT

Nausea, vomiting or diarrhoea	4	observational cohort study	no serious risk of bias	serious	no serious indirectness	no serious imprecision	undetected	none	ÅÅ LOW	IMPORTA NT
Nausea	2	observational cohort study	no serious risk of bias	no serious inconsistency	no serious indirectness	no serious imprecision	undetected	none	ÅÅ LOW	IMPORTA NT
Diarrhoea	4	observational cohort study	no serious risk of bias	no serious inconsistency	no serious indirectness	no serious imprecision	undetected	none	ÅÅ LOW	IMPORTA NT
Altered bowel habits	2	observational cohort study	no serious risk of bias	no serious inconsistency	no serious indirectness	no serious imprecision	undetected	none	ÅÅ LOW	IMPORTA NT
**Neurological symptoms**										
Neurological symptoms	4	observational cohort study	no serious risk of bias	serious	no serious indirectness	no serious imprecision	undetected	none	ÅÅ LOW	IMPORTA NT
Paresthesias	4	observParesthesia sational cohort study	no serious risk of bias	serious	no serious indirectness	no serious imprecision	undetected	none	ÅÅ LOW	IMPORTA NT
**Psychiatric symptoms**										
Sleep difficulty	5	observational cohort study	no serious risk of bias	serious	no serious indirectness	no serious imprecision	undetected	Large magnitude of an effect	ÅÅÅ MODERATE	CRITICAL
Depression	5	observational cohort study	no serious risk of bias	serious	no serious indirectness	no serious imprecision	undetected	Large magnitude of an effect	ÅÅÅ MODERATE	CRITICAL
Anxiety	5	observational cohort study	no serious risk of bias	serious	no serious indirectness	no serious imprecision	undetected	Large magnitude of an effect	ÅÅÅ MODERATE	CRITICAL
PTSD	3	observational cohort study	no serious risk of bias	serious	no serious indirectness	no serious imprecision	undetected	none	ÅÅ LOW	IMPORTA NT
Difficulty concentrating	3	observational cohort study	no serious risk of bias	serious	no serious indirectness	no serious imprecision	undetected	Large magnitude	ÅÅÅ MODERATE	CRITICAL

								of an effect		
					**PFT**					
FVC < 80%	5	observational cohort study	no serious risk of bias	serious	no serious indirectness	no serious imprecision	undetected	none	ÅÅ LOW	IMPORTANT
FEV1 < 80%	3	observational cohort study	no serious risk of bias	serious	no serious indirectness	no serious imprecision	undetected	none	ÅÅ LOW	IMPORTANT
FEV1/FEV < 70%	3	observational cohort study	no serious risk of bias	serious	no serious indirectness	no serious imprecision	undetected	none	ÅÅ LOW	IMPORTANT
VC < 80%	2	observational cohort study	no serious risk of bias	no serious inconsistency	no serious indirectness	no serious imprecision	undetected	none	ÅÅ LOW	IMPORTANT
TLC < 80%	4	observational cohort study	no serious risk of bias	no serious inconsistency	no serious indirectness	no serious imprecision	undetected	none	ÅÅ LOW	IMPORTANT
DLCO < 80%	6	observational cohort study	no serious risk of bias	serious	no serious indirectness	no serious imprecision	undetected	Large magnitude of an effect, confounders likely minimize the effect	ÅÅÅÅ HIGH	CRITICAL
					**CT results**					
CT abnormality	4	observational cohort study	no serious risk of bias	serious	no serious indirectness	no serious imprecision	undetected	Large magnitude of an effect	ÅÅÅ MODERATE	IMPORTANT
GGO	4	observational cohort study	no serious risk of bias	serious	no serious indirectness	no serious imprecision	undetected	none	ÅÅ LOW	IMPORTANT
Consolidation	2	observational cohort study	no serious risk of bias	no serious inconsistency	no serious indirectness	no serious imprecision	undetected	none	ÅÅ LOW	IMPORTANT
Reticular pattern	3	observational cohort study	no serious risk of bias	no serious inconsistency	no serious indirectness	no serious imprecision	undetected	none	ÅÅ LOW	IMPORTANT

Fibrosis	3	observational cohort study	no serious risk of bias	serious	no serious indirectness	no serious imprecision	undetected	none	ÅÅ LOW	IMPORTA NT
Bronchiectasis	2	observational cohort study	no serious risk of bias	serious	no serious indirectness	no serious imprecision	undetected	none	ÅÅ LOW	IMPORTA NT
Nodules	3	observational cohort study	no serious risk of bias	serious	no serious indirectness	no serious imprecision	undetected	Large magnitude of an effect	ÅÅÅ MODERATE	IMPORTA NT
Interlobular spetal thickening	3	observational cohort study	no serious risk of bias	serious	no serious indirectness	no serious imprecision	undetected	none	ÅÅ LOW	IMPORTA NT
Lines and bands	2	observational cohort study	no serious risk of bias	serious	no serious indirectness	no serious imprecision	undetected	Large magnitude of an effect	ÅÅÅ MODERATE	IMPORTA NT
**Quality of life evaluation (EQ-5D-5L)**										
Mobility	2	observational cohort study	no serious risk of bias	no serious inconsistency	no serious indirectness	no serious imprecision	undetected	none	ÅÅ LOW	IMPORTA NT
Personal care	2	observational cohort study	no serious risk of bias	no serious inconsistency	no serious indirectness	no serious imprecision	undetected	none	ÅÅ LOW	IMPORTA NT
Usual activity	2	observational cohort study	no serious risk of bias	serious	no serious indirectness	no serious imprecision	undetected	none	ÅÅ LOW	IMPORTA NT
Pain or discomfort	2	observational cohort study	no serious risk of bias	serious	no serious indirectness	no serious imprecision	undetected	Large magnitude of an effect	ÅÅÅ MODERATE	CRITICAL
Anxiety and depression	2	observational cohort study	no serious risk of bias	serious	no serious indirectness	no serious imprecision	undetected	Large magnitude of an effect	ÅÅÅ MODERATE	CRITICAL
